# Mobile suitcase laboratory for rapid detection of *Leishmania donovani* using recombinase polymerase amplification assay

**DOI:** 10.1186/s13071-016-1572-8

**Published:** 2016-05-13

**Authors:** Dinesh Mondal, Prakash Ghosh, Md Anik Ashfaq Khan, Faria Hossain, Susanne Böhlken-Fascher, Greg Matlashewski, Axel Kroeger, Piero Olliaro, Ahmed Abd El Wahed

**Affiliations:** Center for Nutrition and Food Security, International Center for Diarrheal Disease Research, Bangladesh, Dhaka, Bangladesh; Division of Microbiology and Animal Hygiene, Georg-August-University, Goettingen, Germany; Department of Microbiology and Immunology, McGill University, Montréal, QC Canada; University Medical Centre Freiburg, Centre for Medicine and Society, Freiburg, Germany; UNICEF/UNDP/World Bank/WHO Special Programme for Research and Training in Tropical Diseases (TDR), Geneva, Switzerland; Centre for Tropical Medicine and Global Health, University of Oxford, Oxford, UK

**Keywords:** *Leishmania donovani*, Recombinase polymerase amplification assay, Suitcase laboratory, Visceral leishmaniasis, Bangladesh

## Abstract

**Background:**

*Leishmania donovani* (LD) is a protozoan parasite transmitted to humans from sand flies, which causes Visceral Leishmaniasis (VL). Currently, the diagnosis is based on presence of the anti-LD antibodies and clinical symptoms. Molecular diagnosis would require real-time PCR, which is not easy to implement at field settings. In this study, we report on the development and testing of a recombinase polymerase amplification (RPA) assay for the detection of LD.

**Methods:**

A genomic DNA sample was applied to determine the assay analytical sensitivity. The cross-reactivity of the assay was tested by DNA of *Leishmania* spp. and of pathogens considered for differential diagnosis. The clinical performance of the assay was evaluated on LD positive and negative samples. All results were compared with real-time PCR. To allow the use of the assay at field settings, a mobile suitcase laboratory (56 × 45.5 × 26.5 cm) was developed and operated at the local hospital in Mymensingh, Bangladesh.

**Results:**

The LD RPA assay detected equivalent to one LD genomic DNA. The assay was performed at constant temperature (42 °C) in 15 min. The RPA assay also detected other *Leishmania* species (*L. major*, *L. aethiopica* and *L. infantum*), but did not identify nucleic acid of other pathogens. Forty-eight samples from VL, asymptomatic and post-kala-azar dermal leishmaniasis subjects were detected positive and 48 LD-negative samples were negative by both LD RPA and real-time PCR assays, which indicates 100 % agreement. The suitcase laboratory was successfully operated at the local hospital by using a solar-powered battery. DNA extraction was performed by a novel magnetic bead based method (SpeedXtract), in which a simple fast lysis protocol was applied. Moreover, All reagents were cold-chain independent.

**Conclusions:**

The mobile suitcase laboratory using RPA is ideal for rapid sensitive and specific detection of LD especially at low resource settings and could contribute to VL control and elimination programmes.

**Electronic supplementary material:**

The online version of this article (doi:10.1186/s13071-016-1572-8) contains supplementary material, which is available to authorized users.

## Background

*Leishmania donovani* (LD) causes visceral leishmaniasis (VL) in humans, kala-azar in the Indian Subcontinent (ISC), where a VL elimination programme is underway [1]. Individuals who have been infected through the bite of a sand fly, harbor the parasite in mononuclear phagocytic cells and can remain asymptomatic for the rest of their lives, or develop symptomatic VL. If left untreated, VL could be lethal; after treatment, a proportion of subjects develop a cutaneous form known as post-kala azar dermal leishmaniasis (PKDL).

Diagnosis is currently based on the detection of anti-*Leishmania* antibodies in subjects with clinical symptoms (persisting fever and splenomegaly). Serological assays, e.g. direct agglutination test (DAT) and rK39 dipstick are widely used especially in poor resource settings. DAT is sensitive and specific, but an 8-hour run-time, low reproducibility and challenging quality control are the main drawbacks [2, 3]. In contrast, the rK39 rapid detection assay is very fast (15 min) and easy to use, but its sensitivity and specificity varies [2–6]. In addition, anti-leishmanial antibodies persist for a long time, therefore, current serological tests cannot be used for cure assessment and diagnosis of VL relapse. However, high anti-leishmanial antibody titre before treatment may be useful for prediction of disease progression [7].

The VL elimination programme currently underway in the ISC relies on identifying and treating VL as early and efficiently as possible so as to reduce morbidity and mortality and at the same time remove sources of further transmission [1]. Detection of the parasite or parasite DNA would greatly improve case management, disease control as well as the investigation of transmission dynamics.

The presence of the parasite can be detected through molecular diagnosis using the real-time polymerase chain reaction (PCR), which is highly sensitive and specific [8–12] but unsuited for implementation at primary and secondary health-care facilities. It must be operated in a well-equipped laboratory by highly-trained personnel and reagents must be kept at -20 °C [13].

A highly specific and sensitive test would be needed to sustain the long-term current achievements of the VL elimination programme in the ISC [14]. There is therefore, a demand for a simple and rapid molecular assay. The recombinase polymerase amplification (RPA) assay is an isothermal amplification system [15]. The amplification of the DNA in the RPA relies on enzymes and proteins to replace the repetitive cycles of three temperatures (94 °C, DNA denaturation; 50–60 °C, primer annealing; 72 °C extension) in the PCR. In contrast, the RPA reaction carries out at a constant temperature (42 °C) and even using the body heat [16]. In addition, all reagents are cold chain independent and can be kept at 38–40 °C ambient temperature for up to three months without any effect on the assay performance [17–19].

In this study, RPA assay was developed for the detection of the LD and assay sensitivity, specificity and cross-reactivity were studied. To facilitate the use of the developed assay at point of need, two mobile suitcase laboratories were developed. In addition, operational feasibility of the suitcase laboratory using RPA and SpeedXtract in the field was also explored.

## Methods

### Generation of the DNA LD molecular standard

A molecular DNA standard representing 1–310 nt of LD kinetoplast minicircle DNA (kDNA, GenBank accession number: Y11401.1) was synthesized and inserted into pcDNA3.3-TOPO plasmid vector (GeneArt, Invitrogen, Darmstadt, Germany). The plasmid was linearized with BbSl restriction enzyme (New England Biolabs, Frankfurt am Main, Germany). The number of DNA molecules per microliter was measured by the Quant-iT™ PicoGreen® dsDNA Assay Kit (Fisher Scientific GmbH, Schwerte, Germany) and calculated with an equation as described before [20]. A dilution range of 10^7^–10^1^ molecules/μl of the molecular standard was produced to determine the analytical sensitivity of LD RPA assay. The standard was tested using a real-time PCR assay as described previously [8] applying KAPA SYBR FAST ABI Prism kits (Peqlab, Erlangen, Germany) and on the Stratagene Mx305P device (Agilent, California, USA).

### LD RPA assay primers and probe

To select the RPA primers and exo probe combination, which produces the highest LD RPA assay analytical sensitivity, eight forward primers (FPs), nine reverse primers (RPs) and one exoprobe were tested (Additional file 1: Figure S1). All oligonucleotides were produced by TibMolBiol (Berlin, Germany). The RPA assay was performed by using the TwistAmp exo kits (TwistDx Cambridge, UK) as described below.

### Analytical sensitivity of the LD RPA assay

Concentrations between 10^7^ and 10^1^ molecules/μl of the DNA molecular standard were used to determine the analytical sensitivity of LD RPA assay in eight replicates. In addition, culture promastigote DNA extracted with QIAamp DNA Blood Mini Kit (Qiagen, Hilden, Germany) was used to determine the analytical sensitivity of the RPA assay. Considering 100 fg of culture DNA equivalents to one parasite [21], analytical sensitivity was determined from 100 to 1 parasite, where the volume of template DNA in each reaction was 5 μl.

### Cross-reactivity, clinical sensitivity and specificity of the LD RPA assay

DNA of pathogens listed in Table 2 were used to determine the assay cross-reactivity. Total of 48 archived DNA samples from VL patients, asymptomatic individuals and PKDL patients were tested by both RPA and real-time PCR assays [22]. All VL patients (*N* = 23) were either parasitologically confirmed or diagnosed with VL in accordance with Bangladeshi national guidelines [23]. Likewise, all asymptomatic individuals (*N* = 5) were habitants of VL endemic areas and clinically healthy but positive in Leishmaniasis DAT and rK39 dipstick test. PKDL patients (*N* = 20) were rK39 test positive with previous history of VL and negative for fungus and leprosy. To determine the specificity of the assay, 48 archived DNA samples including endemic healthy controls (*N* = 35), non-endemic healthy controls (*N* = 5) and disease controls (six malaria cases and two TB cases) were also investigated. QIAamp DNA Blood Mini Kit (Qiagen, Hilden, Germany) was used to extract DNA from Buffy coat of VL patients, asymptomatic and control individuals where QIAamp DNA mini kit was used for the skin biopsy from PKDL patients. All VL, PKDL and asymptomatic cases were positive for LD DNA by real-time PCR, while controls (healthy and disease) were negative for rK39, DAT and LD DNA by real-time PCR as described below.

### Real-time PCR for *Leishmania*

Quantitative detection of *Leishmania* DNA was performed on a Biorad CFX96 icycler system using primers and Taqman probe: 5′-GCG ACG TCC GTG GAA AGA A-3′, 5′-GGC GGG TAC ACA TTA GCA GAA-3′ and (FAM): 5′-CAA CGC GTA TTC CC-3′ (Applied Biosystems Inc., Foster City, CA, USA) targeting the repetitive sequence of *L. infantum* Genome (77–142 nt of Genbank accession number: L42486.1) by following a previous method [22]. Each reaction was run using a total of 11 μl of PCR mix (Applied Biosystems Inc., Foster City, CA, USA) plus 9 μl of extracted DNA (buffy coat or skin biopsy). Each sample was run in duplicate. However, samples with very late amplification (≥ 40 cycles) were repeated in triplicate.

### Mobile suitcase laboratory

**Fig. 1:**
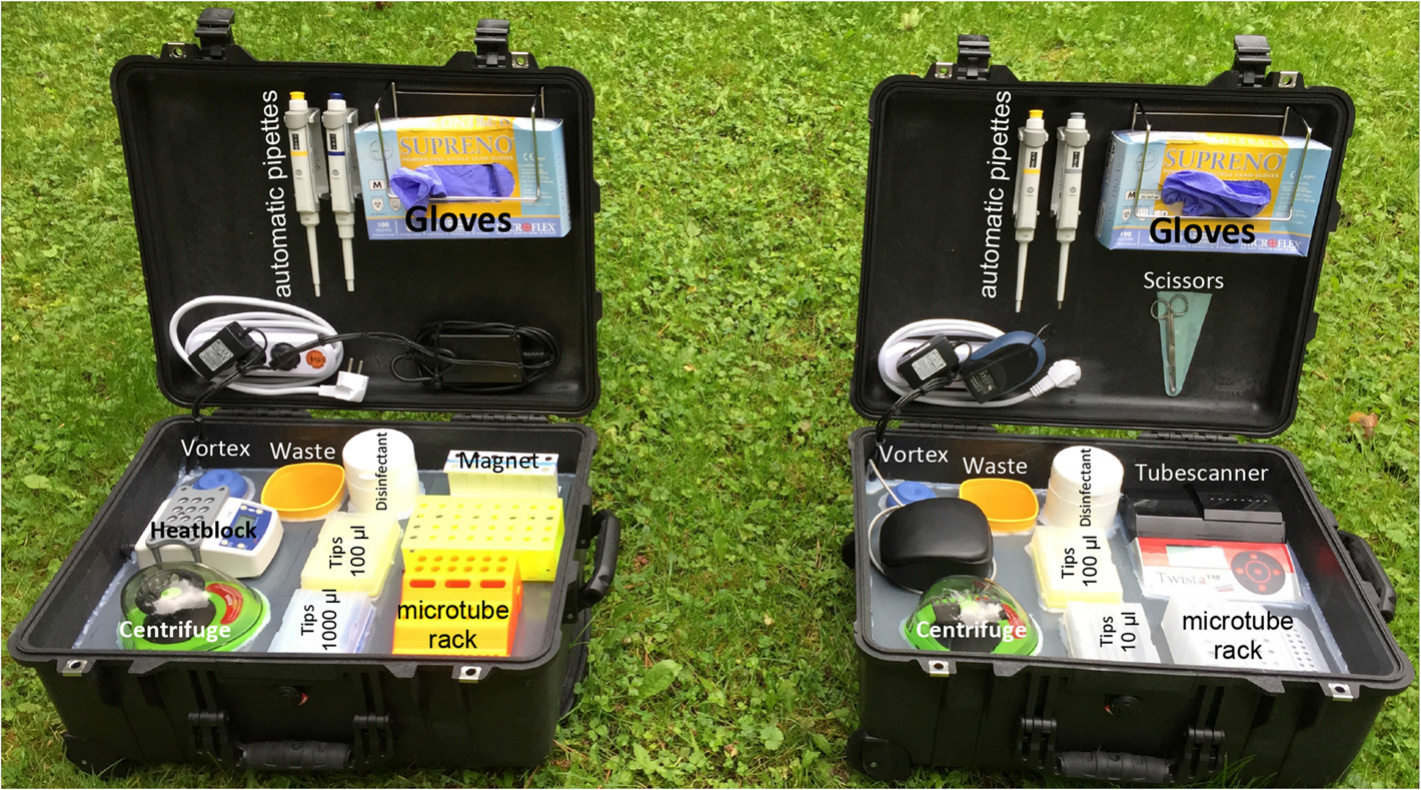
The mobile suitcase laboratories. The mobile set up was built to host all reagents and equipment to perform the SpeedXtract (*left suitcase*). Another suitcase was used to perform the RPA assay (*right suitcase*). The extraction workplace includes in addition to the standard equipment, a heat block and a magnetic stand, while the detection suitcase contains the tubescanner. The size of each suitcase is 56 × 45.5 × 26.5 cm. The bottom of the suitcase was stuffed with foam cubes to absorb shocks. On the top of the foam, a PVC layer was fixed. This PVC layer contained cutouts to host the equipment. All the edges around the equipment and the edges of the case on the PVC layer were glued with hot glue

As described previously [18], two mobile suitcase laboratories (Fig. 1) were constructed to have separate workspaces for nucleic acid extraction and detection in order to avoid any possible contamination. The main idea was to use a water- and dust- resistance case, which was not only employed to transport and store the equipment as well as the reagents, but also to perform the test directly in the suitcase. The mobile set up was powered by solar panels and a power pack (Yeti 400 set, GOALZERO, South Bluffdale, UT, USA). The fully charged battery powers the two laboratories for up to 18 h.

In the extraction suitcase, a simple fast lysis protocol (SpeedXtract, Qiagen, Hilden, Germany) was deployed as follows: 500 μl of whole blood was incubated with 1500 μl of the enrichment buffer and 30 μl of the magnetic beads for 3 min at room temperature. The magnetic beads were separated using a magnetic stand and then the supernatant was removed without disturbing the beads. Then the beads were washed twice with 500 μl enrichment buffer. Thereafter, 100 μl of the lysis buffer was added and the mix was incubated at 95 °C for 10 min. The beads were then separated and 5 μl of the supernatant was used in the RPA reaction. The total time needed for the extraction was 20 min.

In the detection suitcase, 5 μl of the RPA primers and probe mix was added to the RPA lyophilized pellet (TwistAmpexo kits, TwistDx, Cambridge, UK) at concentration of 420 nM and 120 nM, respectively. Then 40 μl of rehydration buffer containing 14 mM Mg acetate was added. Finally, 5 μl of template was added. The tube was closed and mixed well before it was placed into the tubescanner (Twista, TwistDx, Cambridge, UK) and incubated for 15 min at 42 °C. The emitted fluorescence signals were measured at 20 s intervals. A combined threshold and first derivative analysis was used for signal interpretation. The total time for RPA reaction including handling was 20 min.

### Field feasibility evaluation of suitcase laboratory

Blood samples were collected from seven patients hospitalized at the Surya Kanta Kala-azar Research Center in Mymensingh (Additional file 1: Figure S2). Nucleic acid was extracted from whole blood using the SpeedXtrcat method and RPA test was performed in the field as described above. Concurrently, buffy coats were shipped to the central diagnostic laboratory in the icddr, b, where DNA was extracted from the buffy coat using the Qiagen DNA blood kit and real-time PCR was carried out as described before [22].

### Statistical methods

For the LD RPA assay analytical sensitivity using the molecular DNA standard, a semi-log regression analysis and a probit analysis were performed by plotting the RPA threshold time against the number of molecules detected using PRISM (Graphpad Software Inc., San Diego, California) and STATISTICA (StatSoft, Hamburg, Germany), respectively. Sensitivity and specificity were calculated using standard formulas.

### Ethical consideration

The study used archive samples, which were collected through different previous studies with Principal Investigator Dr. Dinesh Mondaland G. M. Khan (PR-13090, PR-13045 and PR-09069). For use of archive samples for eventual future activities on VL, consent from study participants and approval from the Ethical Review Committee of the ​icddr, b had been obtained.

## Results

**Fig. 2:**
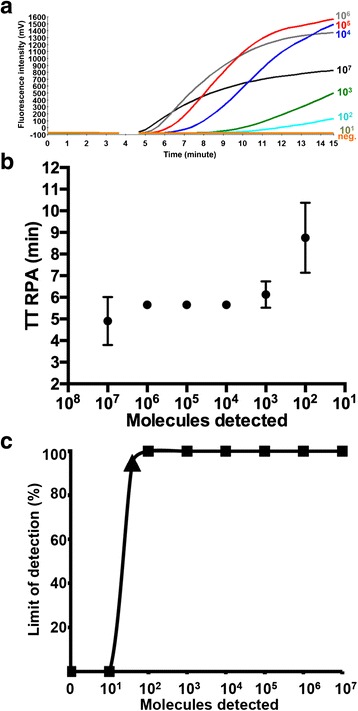
LD RPA assay analytical sensitivity. **a** Fluorescence development in one RPA run by using 10^7^–10^1^ DNA molecules/reaction of the LD DNA molecular standards (Graph generated by ESEquanttubescanner studio software). The sensitivity was 100 DNA molecules. No fluorescence was recorded between 3 and 4 min because a mixing step is necessary to increase the assay sensitivity. **b** Reproducibility of LD RPA assay using data sets of eight RPA assay runs using the DNA molecular standards. LD RPA assay produced results between 3 and 12 min. 10^7^–10^2^ DNA molecules were detected 8 out of 8 runs. 10^1^ copies were not identified by the LD RPA assay. The error bars represent the standard deviation. No error bars were shown for 10^6^–10^4^ because values were consistence at 5.7 min in all eight RPA runs. **c** The probit regression analysis using data of eight RPA assay runs. The limit of detection at 95 % probability (39 DNA molecules) is depicted by a triangle

The analytical sensitivity was determined using 10-fold dilution series of the synthetic molecular kDNA standard (10^7^–10^1^ molecules/μl) and eight FPs, nine RPs and one probe (Additional file 1: Figure S1). Only FP3 + RP3 (Table 1) were able to amplify down to 100 DNA molecules/reaction (Fig. 2a), while the other combinations were not successful.

**Table 1:** LD RPA primers and probe sequences

Name	Sequence (5´-3´)	Amplicon length
FP3	ATGGGCCAAAAACCCAAACTTTTCTGGTCCTC	160 bp
RP3	CTCCACCCGACCCTATTTTACACCAACCCCCAGT	
P	CGCCTCGGAGCCGAT(BHQ1dT)(Tetrahydrofuran)(FAMdT)TGGCATTTTTGGCTATTTTTTGAACGGGAT-phosphate

**Fig. 3:**
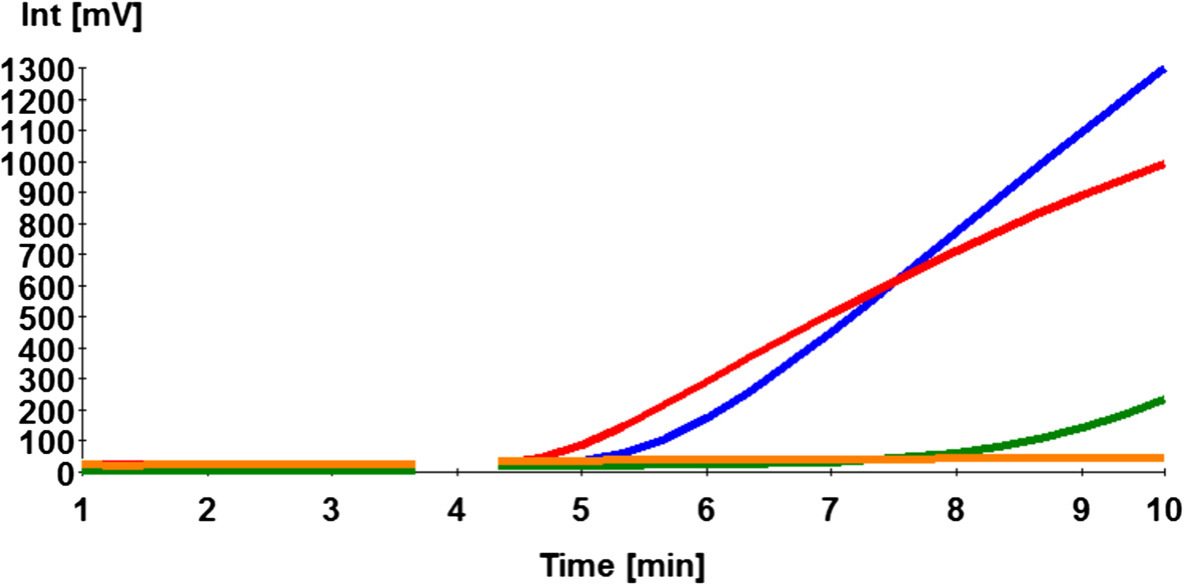
Performance of LD RPA assay on culture promastigote DNA representing with 100 (*red*), 10 (*blue*) and 1 (*green*) LD cell. Limit of detection was one LD cell. *Orange* is the negative control

The FP3 + RP3 were selected for further LD RPA assay validation steps. Eight LD RPA assay runs on the 10^7^–10^1^ dilutions of the molecular standard were performed. The collected data were used in the semi regression and the probit regression analyses. The LD RPA assay results were reproducible in a maximum of 12 min (Fig. 2b). The limit of detection probability in 95 % of cases was 39 DNA molecules (Fig. 2c). Moreover, The LD RPA assay detected down to one LD genomic DNA (Fig. 3).

Standard genomes of LD*, L. major*, *L. aethiopica* and *L. infantum* were positive in the RPA assay, while other *Leishmania* species, parasites and bacteria were negative (Table 2). The sensitivity of the LD RPA assay was estimated using 48 samples from three different forms of the LD infection: VL, asymptomatic and PKDL, whereas another 48 negative control samples were used to determine the specificity of the assay (Table 3 and Additional file 1: Table S1). All samples were tested simultaneously with both LD RPA and real-time PCR assays. The LD RPA assay correctly detected positive samples with 100 % correspondence with the real-time PCR. Moreover, no amplification was detected in the negative samples.

**Table 2:** Cross-reactivity investigation for LD RPA assay

Pathogen name	Number of isolates	Sample type and reference test	LD RPA assay results	Source
*L. donovani* MHOM/ET/67/HU3Z18	1	Reference DNA with concentration of around 40–100 ng/μl	Positive	Paul-Ehrlich-Institute, Langen, Germany
*L. major* MHOM/IL/81/FEBNI	1		Positive	
*L. aethiopica* MHOM/ET/72/L100Z14	1		Positive	
*L. infantum* (50134D) MHOM/TN/80/IPT-1	1		Positive	American Type Culture Collection, Manassas, USA
*L. tropica* (50129D)	1		Negative	
*L. amazonensis*	1		Negative	Dept. of Microbiology, University Medical Center Gottingen, Germany
*L. braziliensis*	1		Negative	
*Toxoplasma gondii*	1		Negative	
*Plasmodium falciparum*	3	DNA extracted from blood samples of malaria patients. Plasmodium species-specific nested PCR was performed [40].	Negative	Icddr, b
*Plasmodium vivax*	3		Negative	
*Salmonella typhi*	1	DNA extracted from culture	Negative	
*Mycobacterium tuberculosis*	2	DNA extracted from lymph node aspirate from two extra pulmonary TB patients. Both were positive in culture and IS6110 PCR [41].	Negative

**Table 3:** Comparison between real-time PCR and LD RPA assays in detecting LD in 96 samples

Subjects	Sample type	Sensitivity (n/N)	Specificity (n/N)
Cases (*N* = 48)			
VL	Buffy coat	100 % (23/23)	NA
Asymptomatic		100 % (5/5)	
PKDL	Skin biopsy	100 % (20/20)	
Controls (*N* = 48)			
Endemic healthy control	Buffy coat	NA	100 % (35/35)
Non-endemic healthy control			100 % (5/5)
Disease control			100 % (8/8)

*Abbreviations: VL* visceral leishmaniasis, *PKDL* post-kala-azar dermal leishmaniasis, *n* number of positives (for sensitivity) or negatives (for specificity), *N* total number of samples, *NA* not applicable

During the field assessment at Surya Kanta Kala-azar Research Centre in Mymensingh (Additional file 1: Figure S2), five samples were positive in both real-time PCR and RPA, while two samples were negative (Additional file 1: Table S2). Using suitcase laboratory, results were produced in 40 min, which included extraction and detection procedures, whereas, real-time PCR was performed in 4 h in a highly equipped laboratory in icddr, b, Dhaka.

## Discussion

The LD RPA assay was designed to amplify 160 nt of the kDNA gene, which is present in a high copy number in LD (~10,000 DNA copies/cell) [24]. The LD RPA assay detected down to 100 DNA copies applying the LD DNA linearized plasmid (Fig. 2), and one LD genomic DNA (Fig. 3). Using 96 buffy coats and skin biopsies collected from VL, asymptomatic and PKDL cases as well as negative control, the LD RPA assays had the same clinical sensitivity and specificity as the real-time PCR [22] (Table 3). However, the RPA produced results six to nine times faster than the real-time PCR, and does not require the same level of equipment and training.

The most challenging step in the RPA assay development is the design of a primer pair able to amplify a very low DNA copy number [13, 17, 18, 25–28]. Neither a program nor strict rules are available. In this study, 8 FPs and 9 RPs were chosen randomly around the exo probe binding sites (Additional file 1: Figure S1). All oligonucleotides combination failed to reach the needed assay sensitivity except FP3 and RP3 (Table 1). The LD RPA exo probe generating the highest fluorescence signal was placed in the same direction as the RP and has a short 5′ end. The same model was successfully applied in two studies [18, 25], but did not performed well in others [13, 19].

The LD RPA assay did detect *L. donovani* and other *Leishmania* spp. (*L. major, L. aethiopica* and *L. infantum*). This is due to the fact that the RPA primers and probe are able to amplify and detect target genes containing 5–9 mismatches [25, 29, 30]. A BLAST search revealed an identity between 93 and 100 % of RPA FP3 and RP3 and 88–100 % of the RPA exo probe to sequences of the above mentioned *Leishmania* species (Additional file 1: Figure S3).

Recently, a RPA assay for the detection of canine VL was developed [31]. The assay amplified both LD and *L. infantum*. It deployed a lateral flow system to readout the results with naked eyes, which tremendously decreases the assay run costs. Nevertheless, this required additional steps to transfer the amplified product to another tube for dilution and detection, which increased the possibility of cross-contamination and the assay run time [32]. In contrast, our approach depended on a probe system that allowed the amplification and the detection in a single closed tube.

Several loop-mediated isothermal amplification (LAMP) assays were established to identify the *Leishmania* parasites [33–38]. The LAMP results can be read by naked eye, nevertheless, the LAMP assay requires six primers and no probe system has yet been implemented. Moreover, LAMP produced results in 60 min [39]. In contrast, the RPA assay was very fast (in a maximum of 15 min) and utilized two primers and one probe.

The SpeedXtract represents a promising tool for the nucleic acid extraction at point of need. First it allowed the isolation of leukocytes from the whole blood in 3 min. Then a simple DNA extraction protocol combining a lysis buffer and heat is applied. Moreover, the reagent is stable at room temperature. The SpeedXtract without the enrichment step was previously deployed for Ebola virus RNA extraction [19]. To the best of our knowledge, this is the first report on using it for a parasitic disease.

There are several reasons why we need a test that can detect the presence of *Leishmania* parasites and can be deployed widely in low-resource settings. Effective case management requires a test that can diagnose VL among other causes of illness (differential diagnosis) and assess as early as possible whether treatment has succeeded or failed (test-of-cure). Effective, sustainable VL control and elimination requires a test that can identify subjects who can transmit the infection as early as possible (even before they become symptomatic) to remove the source of transmission [14]. Especially now that the prevalence of the disease had dropped following the attack phase of the VL elimination programme in the ISC. Such test should be accurate, reproducible, inexpensive and user-friendly.

The current study successfully explored that RPA assay is feasible at field settings to detect leishmaniasis using suitcase laboratory. The selection of suitcase laboratory provided the following advantages: (i) Easy to carry, transport and ship; (ii) Power source from solar panel with power pack; (iii) Easy to be implemented in low resource settings; (iv) A magnetic bead extraction was applied to avoid the creation of aerosols and the use of a high-speed centrifuge; (v) All reagents are cold chain independent; and (vi) A tightened waste container was used, which was autoclaved or incinerated before disposal to avoid contamination to the environment, However, the current cost of the mobile suitcase laboratories and the solar power batteries is 8500 Euro and cost per reaction is six euros inclusive of the controls and the extraction. Lowering the cost will broaden its application in the most affected countries. In addition to diagnosis, integration of an internal positive control and an algorithm for the quantification of number of LD cells will maximize its use as test-of-cure during post-treatment follow-up.

## Conclusion

The use of a mobile suitcase laboratory is advantageous for rapid, sensitive and specific detection of LD using SpeedXtract and RPA assay, especially, at low resource settings such as Bangladesh and could contribute to VL control and elimination program. However, before its recommendation for the program, further validation of the LD-RPA assay incorporated in suitcase laboratory through a prospective study is merited.

## Additional file

Additional file 1: 
**Figure S1**. LD RPA primers and probe sequences aligned with the LD amplicon. One RPA exo probes (P), 8 forward primers (FPs) and 9 reverse primers (RPs) were tested to select combinations yielding the highest analytical LD RPA sensitivity. **Figure S2**. Field exercise in Mymensingh, Bangladesh. (A) The suitcase laboratory, where the nucleic acid extraction using the SpeedXtract kit was performed in 20 min. (B) The RPA assay was accomplished in another suitcase laboratory to avoid cross-contamination. (C) The team while screening blood samples. (D) The research team were able to operate the mobile laboratory during a power cut in the hospital because the laboratory was powered by the solar power pack. **Figure S3**. Mapping 100 sequences derived by BLAST nucleotide search to the LD RPA amplicon as well as RPA primers and probe. The Genbank accession number and the species of *Leishmania* were given. Grey represents the identical sequence. A, C, G, T were highlighted in red, violet, yellow, green, respectively, whenever a mismatch to the LD RPA amplicon was recorded. DNA sequence of *L. donovani, L. infantum, L. major* and *L. chagasi* were picked up by the BLAST search but not other* leishmania* species or nucleotide sequence of other pathogens. **Table S1**. Screening 48 samples with *leishmania* real-time PCR and RPA assays. **Table S2**. Testing clinical samples from patient hospitalized at the Surya Kanta Kala-azar Research Center in Mymensingh, Bangladesh. (DOCX 12654 kb)

## Abbreviations

DAT: direct agglutination test
FP: forward primer
ISC: Indian subcontinent
kDNA: kinetoplast minicircle DNA
LAMP: loop-mediated isothermal amplification
LD: *Leishmania donovani*
n: number of positives (for sensitivity) or negatives (for specificity)
N: total number of samples
NA: not applicable
P: exo probe
PCR: polymerase chain reaction
PKDL: post-kala azar dermal leishmaniasis
RP: reverse primer
RPA: recombinase polymerase amplification
VL: Visceral Leishmaniasis
