# Supplementary material for: Mobile suitcase laboratory for rapid detection of Leishmania donovani using recombinase polymerase amplification assay
Source: Parasit Vectors. 2016 May 13;9:281. doi: 10.1186/s13071-016-1572-8 (PMC4868004; doi:10.1186/s13071-016-1572-8)
Supplement: Additional file 1: — Figure S1. LD RPA primers and probe sequences aligned with the LD amplicon. One RPA exo probes (P), 8 forward primers (FPs) and 9 reverse primers (RPs) were tested to select combinations yielding the highest analytical LD RPA sensitivity. Figure S2. Field exercise in Mymensingh, Bangladesh. (A) The suitcase laboratory, where the nucleic acid extraction using the SpeedXtract kit was performed in 20 min. (B) The RPA assay was accomplished in another suitcase laboratory to avoid cross-contamination. (C) The team while screening blood samples. (D) The research team were able to operate the mobile laboratory during a power cut in the hospital because the laboratory was powered by the solar power pack. Figure S3. Mapping 100 sequences derived by BLAST nucleotide search to the LD RPA amplicon as well as RPA primers and probe. The Genbank accession number and the species of Leishmania were given. Grey represents the identical sequence. A, C, G, T were highlighted in red, violet, yellow, green, respectively, whenever a mismatch to the LD RPA amplicon was recorded. DNA sequence of L. donovani, L. infantum, L. major and L. chagasi were picked up by the BLAST search but not other leishmania species or nucleotide sequence of other pathogens. Table S1. Screening 48 samples with leishmania real-time PCR and RPA assays. Table S2. Testing clinical samples from patient hospitalized at the Surya Kanta Kala-azar Research Center in Mymensingh, Bangladesh. (DOCX 12654 kb) [file 13071_2016_1572_MOESM1_ESM.docx]

>Amplicon_RPA ATGGGCCAAAAACCCAAACTTTTCTGGTCCTCCGGGTAGGGGCGTTCTGCGAAAACCGAAAAATGGGTGCAGAAATCCCGTTCAAAAAATAGCCAAAAATGCCAAAAATCGGCTCCGAGGCGGGAAACTGGGGGTTGGTGTAAAATAGGGTCGGGTGGAGGGGAAATTCGGGGCTCGGACGTGTGTGGATATGGCCTGGGTGGGGACT

>LD_RPA_FP1 -----------------------------------------GCGTTCTGCGAAAACCGAAAAATGGGTGCAGA---------------------------------------------------------------------------------------------------------------------------------------

>LD_RPA_FP2 --------------CAAACTTTTCTGGTCCTCCGGGTAGGGGCGT-------------------------------------------------------------------------------------------------------------------------------------------------------------------

>LD_RPA_FP3 ATGGGCCAAAAACCCAAACTTTTCTGGTCCTC--------------------------------------------------------------------------------------------------------------------------------------------------------------------------------

>LD_RPA_FP4 -----------------------CTGGTCCTCCGGGTAGGGGCGTTCTGCGA------------------------------------------------------------------------------------------------------------------------------------------------------------

>LD_RPA_FP5 ---------------------------------------------TCTGCGAAAACCGAAAAATGGGTGCAGA---------------------------------------------------------------------------------------------------------------------------------------

>LD_RPA_FP6 -----------------ACTTTTCTGGTCCTCCGGGTAGGGGCGT-------------------------------------------------------------------------------------------------------------------------------------------------------------------

>LD_RPA_FP7 ATGGGCCAAAAACCCAAACTTTTCTG--------------------------------------------------------------------------------------------------------------------------------------------------------------------------------------

>LD_RPA_FP8 ATGGGCCAAAAACCCAAACTTTTCTGGTCCTCCG------------------------------------------------------------------------------------------------------------------------------------------------------------------------------

>LD_RPA_P_rc --------------------------------------------------------------------------ATCCCGTTCAAAAAATAGCCAAAAATGCCANNNATCGGCTCCGAGGCG--------------------------------------------------------------------------------------

>LD_RPA_RP1_rc------------------------------------------------------------------------------------------------------------------------------------------TGTAAAATAGGGTCGGGTGGAGGGGAAATTCG--------------------------------------

>LD_RPA_RP2_rc------------------------------------------------------------------------------------------------------------------------------------------------------------------------------TCGGACGTGTGTGGATATGGCCTGGGTGGGGACT

>LD_RPA_RP3_rc------------------------------------------------------------------------------------------------------------------------------ACTGGGGGTTGGTGTAAAATAGGGTCGGGTGGAG------------------------------------------------

>LD_RPA_RP4_rc-------------------------------------------------------------------------------------------------------------------CGGGGCGGGAAACTGGGGGTTGGTGTAAAATAG------------------------------------------------------------

>LD_RPA_RP5_rc-------------------------------------------------------------------------------------------------------------------------------------------------------------GAGGGGAAATTCGGGGCTCGGACGTGTGTGGATA-----------------

>LD_RPA_RP6_rc-----------------------------------------------------------------------------------------------------------------------------------------------------GTCGGGTGGAGGGGAAATTCGGGGCTCG-------------------------------

>LD_RPA_RP7_rc-------------------------------------------------------------------------------------------------------------------------------------GTTGGTGTAAAATAGGGTCGGGTGGAG------------------------------------------------

>LD_RPA_RP8_rc------------------------------------------------------------------------------------------------------------------------------ACTGGGGGTTGGTGTAAAATAGGGTCG-------------------------------------------------------

>LD_RPA_RP9_rc-------------------------------------------------------------------------------------------------------------------------------------GTTGGTGTAAAATAGGGTCGGGTG---------------------------------------------------

**Fig S1. LD RPA primers and probe sequences aligned with the LD amplicon.** One RPA exo probes (P), 8 forward primers (FP), and 9 reverse primers (RP) were tested to select combinations yielding the highest analytical LD RPA sensitivity. NNN are sites of the quencher and fluorophore in following order (BHQ1-dT) (Tetrahydrofuran) (FAM-dT). rc is the reverse complementary of the original sequence used in the experiment.

**Fig S2. Field exercise in Mymensingh, Bangladesh.** (A) The suitcase laboratory, where the nucleic acid extraction using the SpeedXtract kit was performed in 20 minutes. (B) The RPA assay was accomplished in another suitcase laboratory to avoid cross-contamination. (C) The team while screening blood samples. (D) The research team were able to operate the mobile laboratory during a power cut in the hospital because the laboratory was powered by the solar power pack.

**Fig S3. Mapping 100 sequences derived by BLAST nucleotide search to the LD RPA amplicon as well as RPA primers and probe.** The alignment was performed by using Geneious (V: 9.0.5, Biomatters Limited, New Zealand). The Genbank accession number and the species of *Leishmania* were given*.* Grey represents the identical sequence. A, C, G, T were highlighted in red, violet, yellow, green, respectively, whenever a mismatch to the LD RPA amplicon was recorded. DNA sequence of *L. donovani, L. infantum, L. major and L. chagasi* were picked up by the BLAST search but not other *leishmania* species or nucleotide sequence of other pathogens.

**Table S1. Screening 48 samples with *leishmania* real-time PCR and RPA assays.** All VL patients (N=23) and all asymptomatic individuals (N=5) were positive in Leishmaniasis DAT and rK39 dipstick test. PKDL patients (N=20) were rK39 test positive with previous history of VL.

|  | **ID** | **Sample**  **type** | **RPA**  **(TT- min)** | **Real-time PCR**  **(CT Value)** |
| --- | --- | --- | --- | --- |
| Visceral Leishmaniasis | WBA04 | Buffy Coat | 5.7 | 32.14 |
|  | WBA05 |  | 5.7 | 32.18 |
|  | WBA06 |  | 5.7 | 35.96 |
|  | WBA07 |  | 5.7 | 34.17 |
|  | WBA08 |  | 6.0 | 35.23 |
|  | Apu 87 |  | 6.0 | 34.44 |
|  | Apu 89 |  | 6.0 | 34.2 |
|  | Apu 93 |  | 6.0 | 32.25 |
|  | Apu 95 |  | 6.0 | 32.19 |
|  | Apu 99 |  | 5.7 | 34.5 |
|  | Apu 102 |  | 5.7 | 35.08 |
|  | Apu 121 |  | 5.7 | 34.01 |
|  | Apu 122 |  | 5.7 | 34.83 |
|  | Apu 123 |  | 5.7 | 34.06 |
|  | Apu 128 |  | 5.7 | 32.75 |
|  | Apu 129 |  | 5.7 | 33.24 |
|  | Apu 132 |  | 5.7 | 33.16 |
|  | Apu 106 |  | 6 | 39.53 |
|  | Apu 101 |  | 6.7 | 38.24 |
|  | Apu 124 |  | 5.7 | 37.73 |
|  | Apu 109 |  | 7 | 38.69 |
|  | Apu 90 |  | 5.3 | 34.12 |
|  | Apu 110 |  | 5.3 | 39.71 |
| Asymptomatic | Rob-298 |  | 6.3 | 34.85 |
|  | Rob-3264 |  | 8.3 | 37.1 |
|  | Rob-3431 |  | 8 | 37.15 |
|  | Rob-8052 |  | 5.7 | 34.5 |
|  | Rob-3070 |  | 5.7 | 34.07 |
| PKDL | TRF 89 | Skin biopsy | 5.3 | 29.27 |
|  | TRF 44 |  | 5.3 | 29.59 |
|  | TRF 96 |  | 6 | 37.82 |
|  | TRF103 |  | 7.3 | 39.56 |
|  | TRF 83 |  | 7.7 | 37.57 |
|  | TRF 92 |  | 6 | 37.7 |
|  | TRF 52 |  | 5.7 | 33.63 |
|  | TRF 58 |  | 5.7 | 35.43 |
|  | TRF 85 |  | 5.7 | 34.37 |
|  | TRF 93 |  | 5.7 | 38.56 |
|  | TRF 95 |  | 5.7 | 33.01 |
|  | TRF 97 |  | 7.3 | 37.46 |
|  | TRF 91 |  | 9 | 38.62 |
|  | TRF 60 |  | 6.7 | 34.88 |
|  | TRF 88 |  | 7.3 | 37.22 |
|  | TRF102 |  | 5.7 | 33.93 |
|  | TRF100 |  | 8.3 | 37.84 |
|  | TRF126 |  | 5.7 | 27.29 |
|  | TRF119 |  | 5.7 | 37.43 |
|  | TRF116 |  | 2.3 | 35.91 |

PKDL, Post-kala-azar dermal leishmaniasis;

TT, threshold time in minutes; CT, cycle threshold.

**Table S2. Testing clinical samples from patient hospitalized at the Surya Kanta Kala-azar Research Center in Mymensingh, Bangladesh.**

| **Sample**  **ID** | **Type** | **SE + RPA**  **(TT in min)** | **QE + real-time PCR**  **(Ct)** |
| --- | --- | --- | --- |
| B7 | VL | 5.7 | 39.24 |
| B6 | CL | neg | neg |
| B1 | VL | 6.3 | 38.41 |
| B8 | VL | 5.7 | 38.72 |
| B4 | PKDL | neg | neg |
| B10 | VL | 7.0 | 34.53 |
| B5 | VL | 8.0 | 35.38 |

VL is the visceral leishmaniasis; CL, cutaneous leishmaniasis; PKDL, Post-kala-azar dermal leishmaniasis; SE, the SpeedXtract method; QE, Qiagen DNA blood extraction kit; TT, threshold time in minutes; Ct, cycle threshold.
